# An Examination of the Practice Approaches of Canadian Dietitians Who Counsel Higher-Weight Adults Using a Novel Framework: Emerging Data on Non-Weight-Focused Approaches

**DOI:** 10.3390/nu15030631

**Published:** 2023-01-26

**Authors:** Kori Lichtfuss, Beatriz Franco-Arellano, Jennifer Brady, JoAnne Arcand

**Affiliations:** 1 Faculty of Health Sciences, Ontario Tech University, Oshawa, ON L1G 0C5, Canada; 2 School of Nutrition and Dietetics, Acadia University, Wolfville, NS B4P 2R6, Canada

**Keywords:** non-weight-focused approaches, Registered Dietitians, Canada, obesity clinical practice guidelines

## Abstract

Non-weight-focused approaches (NWFAs) may be used by some clinicians when working with higher-weight clients. In contrast to weight-focused approaches (WFAs), NWFAs de-emphasize or negate weight loss and emphasize overall diet quality and physical activity. The extent to which WFAs, NWFAs, or a combination of both WFAs and NWFAs are used by dietitians is unknown in Canada and globally. This study surveyed Canadian Registered Dietitians (RDs) who counsel higher-weight clients to assess which practice approaches are most commonly used, how they view the importance of weight, and how they define “obesity” for the study population. Five practice approaches were initially defined and used to inform the survey: solely weight-focused; moderately weight-focused; those who fluctuate between weight-focused/weight-inclusive approaches (e.g., used both approaches); weight inclusive and; weight liberated. Participants (n = 383; 94.8% women; 82.2% white) were recruited using social media and professional listservs. Overall, 45.4% of participants used NWFAs, 40.5% fluctuated between weight-focused/moderately weight-focused, and 14.1% used weight-focused approaches (solely weight focused and moderately weight focused). Many participants (63%) agreed that weight loss was not important for higher-weight clients. However, 81% of participants received no formal preparation in NWFAs during their education or training. More research is needed to understand NWFAs and to inform dietetic education in support of efforts to eliminate weight stigma and provide inclusive access to care.

## 1. Introduction

Nutrition therapy from a dietitian is recommended for higher-weight clients, traditionally classified as “overweight” and “obese”, alongside lifestyle, medical, and surgical interventions [1]. The recent Canadian Adult Obesity Clinical Practice Guidelines (CPGs) included weight-loss-centred recommendations that were accompanied by fourteen evidence-based dietary pattern and/or food-based strategies to induce weight loss were endorsed, as well as included non-diet approaches [2]. Non-diet approaches typically emphasize overall diet quality and physical activity and de-emphasize weight loss. The variation among these recommendations reflects the range of dietetic practice approaches, as well as the diverse perspectives of dietitians on if and how “obesity” should be addressed.

Traditionally, nutrition therapy for higher-weight individuals has taken a weight-centric or uses weight-focused approaches (WFAs) in which calorie restriction and physical activity are encouraged to induce weight loss [3,4]. This perspective considers that individuals have control over their body size, which underplays the myriad social, physiological, and environmental influences that impact body weight. Unfortunately, the use of WFAs is associated with weight stigma, which has deleterious psychological effects [5,6] that contribute to the avoidance of health-promoting behaviours, and increased stress, anxiety, depression and suicidal ideation among higher-weight individuals [7].

In contrast, weight-inclusive, non-diet, or non-weight-focused approaches (NWFAs) have become increasingly common approach used by dietitians when working with higher-weight clients. Nutrition therapy techniques used by dietitians practicing with NWFAs may include intuitive eating, mindful eating, Health at Every Size ^®^ (HAES^®^), and other non-restrictive approaches. Some dietitians have moved towards NWFAs in light of studies showing that weight-loss interventions do not consistently yield sustainable, long-term weight changes or improved health [8,9]. They also recognize that NWFAs are associated with reduced morbidity regardless of weight loss or body mass index [10,11,12,13]. Finally, some consider NWFAs to be more client centred because they are believed to redress weight stigma [3,14]. Some of those using NWFAs reject the use of the term “obesity” due to its questionable scientific validity, its strong negative connotations that can contribute to weight stigma, and the fact that people in higher weights generally do not prefer it [1,4,15]. NWFAs (“non-dieting approach”) appears in the Canadian Adult Obesity CPGs recommendations, which to our knowledge is the first set of obesity-related CPGs to do so [16].

There is little contemporary literature that describes the approaches that dietitians take when working with higher-weight clients [17], and a scarcity of data related to NWFAs. Among dietitians who use NWFAs, it is likely that the nutrition therapy approaches and techniques they use are highly variable, since no practice guidelines on the matter exist and dietetic curricula in Canada rarely include education or training in NWFAs. Only one known study from Australia has examined the diverse approaches dietitians use with respect to body weight, while other studies have examined dietitians’ use of a single practice approach [3,17,18,19]. This is a significant research gap given the recent addition of non-diet approaches to the Canadian Adult Obesity CPGs, the apparent growing popularity of NWFAs, and the seeming limited education and training that dietitians receive in NWFAs [4,20].

The objective of this study was to describe the practice approaches and perspectives of Canadian dietitians who counsel higher-weight adult clients. We first developed and validated a classification framework comprising five practice approach types. Practice approach classifications were defined by the extent to which a dietitian focuses on weight loss, as an important outcome of the nutrition therapy when working with higher-weight adult clients. We then administered a survey to assess the proportion of dietitians who practice according to each of the practice approach types, and how the views, nutrition therapy techniques, and education and training backgrounds among respondents differ across the spectrum of approaches used.

## 2. Materials and Methods

### 2.1. Study Design

This study used a cross-sectional design that included an online survey of Canadian Registered Dietitians (RDs). The online survey was developed in two phases: (1) development and validation of the practice approach classification framework; (2) development, validation, and pilot testing of the full survey. Each phase is described in detail below. The survey was disseminated nationally using QualtricsXM (Provo, UT, USA) and was open from May to July 2021. This study was reviewed by the Ontario Tech University Research Ethics Board (File #16078).

### 2.2. Development and Validation of the Practice Approach Classifications

A framework was developed to classify the spectrum of practice approaches used by dietitians. Practice approaches were defined by the extent to which a dietitian focuses on weight loss as an important outcome of the nutrition therapy they deliver. The research team used an iterative, consultative approach to derive the classifications, which began with a review of the literature and the development of an initial set of practice approach categories with corresponding definitions [3,4]. Next, ten clinical dietitians who used various practice approaches, and nutrition scientists with experience in survey development, reviewed the initial classification framework and participated in a focus group to provide insights into the proposed approaches. Extra care was taken to ensure that the language in the questions used to guide the focus group did not suggest a preference among the research team for any of the practice approach categories (e.g., using BMI instead of higher-weight client would likely produce response bias among either approach). Afterwards, the classification framework was revised to integrate the feedback obtained during the focus group. The revised classification framework was iteratively re-reviewed and revised by the same dietitians and nutrition scientists until consensus was reached. Finally, a larger pool of clinical dietitians who use various practice approaches was engaged to further refine the classification framework. Table 1 lists the final practice approach categories and their descriptive criteria that informed the online survey.

In the text of the online survey, the five practice approach categories were labelled A to E to avoid biasing responses, which may have arisen if suggestive labels were used to identify each. When the survey was administered, participants were asked to self-classify their practice approach based on the descriptive criteria. An open-ended response question asked participants to identify one or more labels for what they call their practice approach. Hereafter, the term “weight-focused” is used to refer to the solely weight-focused and moderately weight-focused approaches categories, and “NWFAs” is used to refer to the weight inclusive and weight-liberated practice approach categories.

### 2.3. Development, Validation, and Pilot Testing of the Full Survey

The questionnaire was developed based on published literature [17] and the Consolidated Framework for Implementation Research [21]. The same group of clinical dietitians and nutrition scientists (n = 10) who participated in the validation of the practice approach classification framework, also participated in the face and content validation and pilot testing of the full questionnaire. A validation survey was developed by adapting sixteen questions from Simon and White’s survey [22], and included open-ended comments and ratings on a 5-point Likert scale (e.g., 1 = strongly disagree, 3 = neither agree nor disagree, 5 = strongly agree). Questions explored multiple components of survey development including whether the survey questions were direct, ambiguous, leading, relevant, and necessary. Changes were made to the survey based on the feedback obtained. Later, the same reviewers pilot tested the online survey and provided additional feedback on the acceptability of the online format of the questionnaire. The research team also ensured the data produced were free of errors and are usable.

The final survey questionnaire included a set of demographic questions (n = 11); a question asking the participant to classify themselves into one of five practice approaches used when working with higher-weight adults (n = 1); questions to determine dietitians’ overall perceptions of the importance of weight loss (n = 1); definitions of obesity (n = 1); a set of “select all that apply” for questions related to nutrition assessment methods used, and the nutrition therapy approaches and nutrition therapy techniques most commonly applied (n = 47).

### 2.4. Participants and Recruitment

To be included in this study, participants had to be a member of a provincial dietetic regulatory body and currently be providing nutrition therapy to higher-weight adults in an outpatient setting. Undergraduate, graduate, and practicum students and retired dietitians were excluded.

Purposive sampling was used to recruit participants by circulating a survey invitation via email distribution lists of dietitians and via advertisements posted to dietetic professional social media groups (e.g., Facebook). The email distribution lists were operated by national and regional dietetic professional organizations such as Dietitians of Canada, Dietitians of Canada practice-based member networks (e.g., Consulting dietitians; Diabetes, Obesity and Cardiovascular networks), Obesity Canada, the registrant lists of five provincial dietetic regulatory bodies, and other independently operated email listservs (e.g., Gerry’s list). Participants were encouraged to share the survey link to maximize snowball sampling and reach those who are not members of the aforementioned platforms. To maximize reach, dietitians who may have been out of the office, the tailored Dillman’s design method was followed and circulated survey invitation reminders every few weeks [23,24]. To incentivize participation, participants were offered the opportunity to enter a draw to win one of ten $75 CAD Amazon gift cards. A minimum sample size of 230 participants was needed to make inferences, based on data from Willer et al. (2019) where 18.3% of participants used weight-neutral practice approaches, and considering an alpha level of 0.05 and a power at 80%.

### 2.5. Analysis

Data were cleaned and analyzed using RStudio. There were 855 survey responses, 472 bot responses were identified by cross-referencing the province stated and validating with GPS coordinates using Google maps (any responses outside of Canada were assumed to be from bots and were excluded). There were no incomplete surveys, defined as <5% questions unanswered. The final sample included 383 responses. Frequencies and percentages were calculated for categorical variables. Between-group characteristics were analyzed with chi-square tests for categorical variables, with Fishers’ exact test when cell counts were <5. We also compared “Yes” versus “No” responses for each variable. A *p*-value <0.05 was considered statistically significant.

## 3. Results

Participants were 94.8% women and 82.2% White (Table 2). One-third of participants resided in Ontario or Alberta and had been practicing as an RD for 0–5 years. Approximately half of participants practiced in primary care (50.9%) and in an urban setting (54.3%).

### 3.1. Practice Approaches Used and Views

**Figure 1:**
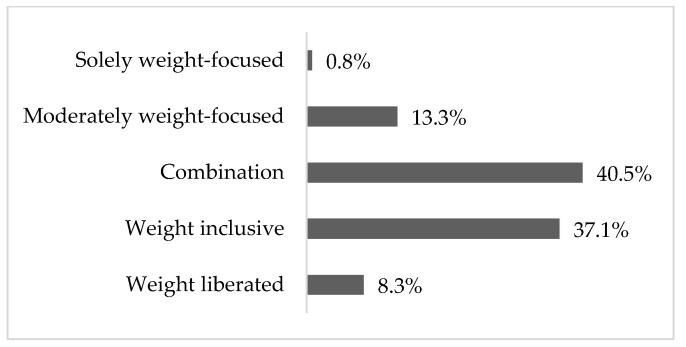
The proportion of Registered Dietitians who practice using the following approaches when working with higher-weight clients.

NWFAs were used by 45.4% of participants (37.1% weight inclusive; 8.3% weight liberated), 40.5% used a combined approach (those that fluctuate between weight-focused and weight inclusive practice approaches), and 14.1% used weight-focused approaches (0.8% solely weight focused and 13.3% moderately weight focused) (Figure 1). Table 3 shows how participants classified themselves into a practice approach. Approximately half of the participants who worked in primary care used NWFAs (52.3%) (Table 2). Of those who ascribed to weight-focused approaches, more participants were from Alberta (21.6%, *p* = 0.037) and worked in an outpatient setting (23.0%, *p* < 0.001).

Overall, 88% of participants indicated that their current practice reflected their preferred approach either most of the time or all of the time. Alignment between current and preferred practice approach differed by practice approach category: 100% for those using weight-liberated approaches or solely weight focused; 90.9% for those using a weight-inclusive approach; 85.2% for those using a combined approach; 82.3% for those using moderately weight focused (*p* = 0.007). The ways in which participants defined “obesity”, according to practice type, are presented in Table 4, by selecting a pre-set response. The majority of those who used WFAs considered “obesity” to be “a complex and progressive chronic disease” (solely weight focused 66.7%, n = 2/3; moderately weight focused 62.7%, n = 32/51) and “characterized by abnormal, excessive body fat (adiposity) that impairs health” (solely weight focused 66.7%, n = 2/3; moderately weight focused 70.6%, n = 36/51). In contrast, participants who used weight-inclusive approach (46.5%, n = 66/142) and weight-liberated approach (87.5%, n = 28/32) did not recognize the word “obesity” nor use the “obesity” language definition.

### 3.2. Practice Techniques Used When Working with Higher-Weight Clients

Table 5 reports the nutrition assessment methods, and nutrition therapy approaches and techniques that participants used overall and across each of the five practice approaches. When conducting dietary assessments most participants reported monitoring general health behaviours (95.8%), as well as metabolic parameters, such as lipid profile or blood glucose (95.6%), mental health status (94.0%), and social indicators of health (91.4%). A high proportion of participants recommended similar nutrition therapy approaches, such as increasing fruit and vegetables (95.0%), whole grains (88.3%), increasing diet variety (86.9%) and pulses (85.6%). Fewer participants recommended replacing saturated/trans fats with unsaturated fats (64.2%) or the use of specific dietary patterns such as the Mediterranean diet (55.1%), Canada’s Food Guide (53.8%), a low-glycemic diet (41.8%), or the DASH diet (33.4%). The most common nutrition therapy techniques used comprised principles of mindful eating (88.0%), recognizing adult clients’ lived experiences (79.4%), discussions with adult clients about the structural barriers to their being/feeling healthy or wellness (75.5%), intuitive eating principles (70.8%), compassion-informed nutrition therapy strategies (65.3%), and Health At Every Size^®^ principles (64.8%). In contrast, the least common nutrition therapy techniques used were explicitly recommending weight loss (5.2%), keeping a food diary (3.9%), and commercial weight loss products (0.3%).

### 3.3. Summary of the Practice Approach Categories Used by Participants

The proceeding sections describe the practice approach categories used by participants when working with higher-weight adult clients.

#### 3.3.1. Weight-Focused Approaches

Weight-focused approaches comprise of both solely weight-focused and moderately weight-focused practice approach categories. Intentional weight loss was rated as being important or very important by 100% (n = 3/3) of participants who practiced using solely weight-focused approaches and 33.3% (17/51) of those who practiced using moderately weight-focused approaches (*p* < 0.001). A higher proportion of those who used a solely weight-focused approach (66.7%, n = 2/3) recommended weight loss compared to those who used a moderately weight-focused approach (23.5%, n = 12/51; *p* < 0.001). Compared to all practice types, solely weight-focused participants more often recommended alternative foods for snacking and replacing saturated/trans fats with unsaturated fats (100%, n = 3/3, *p* = 0.004), and for both WFA participants more often recommended foods to reduce calories (solely weight focused 100%, n = 3/3; moderately weight focused 62.7%, n = 32/51; *p* < 0.001) (Table 5). Weight-focused approaches also more often weighed clients (solely weight focused 100%, n = 3/3; moderately weight focused 84.3%, n = 43/51; *p* < 0.001) and calculated BMI (solely weight focused 66.7%, n = 2/3; moderately weight focused 80.4%, n = 41/51; *p* < 0.001) when compared to all practice approaches. A higher proportion of weight-focused participants compared to all other practice approaches also recommended keeping a food intake dairy (solely weight focused 100%, n = 3/3; moderately weight focused 72.5%, n = 37/51; *p* < 0.001) and limiting snacking (solely weight focused 66.7%, n = 3/3; moderately weight focused 49.0%, n = 25/51; *p* < 0.001), compared to all other practice approaches.

#### 3.3.2. Characteristics of Combined Approaches

Participants who practiced using a combination approach fluctuated between weight-focused and weight-inclusive approaches. Only a small proportion (6.5%, n = 10/155) of these participants reported believing intentional weight loss to be important, and only 3.9% (n = 6/155) reported regularly recommending weight loss (*p* < 0.001), which was lower than all the other practice approaches (*p* < 0.001 for each variable). These participants also more often recommended increasing the intake of pulses (91.0%, n = 141/155; *p* < 0.001) and using Canada’s Food Guide (57.4%, n = 89/155; *p* = 0.018) compared to the other practice approaches. In contrast, a lower proportion of these participants recommended eating fewer calories (13.5%, n = 21/155; *p* < 0.001), reducing total fat intake (13.5%, n = 21/155; *p* = 0.003) and keeping a weight diary (1.9%, n = 3/155; *p* < 0.001) when compared to the other practice approaches.

#### 3.3.3. Characteristics of Non-Weight-Focused Approaches

Intentional weight loss was rated as not very important/not important among many dietitians who use weight-inclusive approaches (83.1%, n = 118/142) and weight-liberated approaches (87.5%, n = 28/32), when compared to all the other practice approaches (*p* < 0.001). No participants who used NWFAs recommended weight loss to clients, yet 19.7% (n = 28/142) of weight inclusive and 15.6% (n = 5/32) of weight liberated participants reported weighing clients. Additionally, the majority of those who practiced NWFAs monitor health behaviours, e.g., diet and exercise (weight inclusive 97.0%, n = 138/142; weight liberated 84.4%, n = 27/32; *p* = 0.048). A large proportion reported typically assessing clients’ metabolic parameters, e.g., lipid profile, blood glucose (weight inclusive 96.5%, n = 137/142; weight liberated 87.5%, n = 28/32) and mental health status (weight inclusive 94.4%, n = 134/142; weight liberated 96.9%, n = 31/32). Some participants indicated that assessment techniques they use were not included in the survey. Among participants using a weight-inclusive approach, 14.8% (n = 21/142) indicated that they also used “Other” nutrition therapy assessment techniques that they described as weighing on the client’s request, assess occupation status, sleep hygiene and body image. Among those using weight-liberated approaches, 28.1% (n = 9/32) indicated that they also used “Other” nutrition therapy assessment techniques that they labelled as assessing body image, spiritual values and health values.

Compared to other practice approaches, the most common dietary approaches used among dietitians using NWFAs were: increasing fruits and vegetables (weight inclusive 97.2%, n = 138/142; weight liberated 81.3%, n = 26/32; *p* = 0.014), suggest increasing intake of whole grains (weight inclusive 91.5%, n = 130/142; weight liberated 75.0%, n = 24/32; *p* < 0.001), increasing diet variety (weight inclusive 89.4%, n = 127/142; weight liberated 90.6%, n = 29/32; p0.215) and increasing intake of pulses (weight inclusive 88.0%, n = 125/142; weight liberated 78.1%, n = 25/32; *p* < 0.001). Of those who used a weight-inclusive approach, 21.8% (n = 31/142) indicated that they recommend “Other” nutrition therapy approaches that participants labelled as personalizing recommendations on a client-by-client basis, regular/consistent meal patterns, mechanical eating, holistic, traditional Indigenous eating patterns, mindful and intuitive eating and also some preferred not to label the pattern or did not recommend but rather allowed the client to guide. Finally, of those using weight-liberated approaches, 43.8% (n = 14/32) indicated that they recommend “Other” nutrition therapy approaches not included on the questionnaire, such as guided by client’s needs and health values, gentle nutrition, inclusion approach (not restriction), and base on foods client is familiar with and have access to.

The majority of participants reported that lived experiences impact the lives of adult clients in ways that are often hidden to health care providers (weight inclusive 87.3%, n = 124/142; weight liberated 96.9%, n = 31/32; *p* < 0.001), and more commonly used in their practice the principles of intuitive eating (weight inclusive 87.3%, n = 124/142; weight liberated 93.8%, n = 30/32; *p* < 0.001) and Health At Every Size^®^ (weight inclusive 85.2%, n = 121/142; weight liberated 90.6%, n = 29/32; *p* < 0.001), compared to all other approaches. Of those who used weight-inclusive approaches, 7.0% (n = 10/142) indicated that they also used “Other” nutrition therapy practice approaches that they labelled as motivational interviewing, encourage clients to not weigh themselves and use a chronic disease self-management approach. Of those who used weight-liberated approaches, 12.5% (n = 4/42) indicated that they also used “Other” nutrition therapy practice approaches that they labelled as eating competence, acceptance and commitment therapy (ACT) and encourage clients to not weigh themselves.

### 3.4. Education and Training for Non-Weight-Focused Approaches

The majority of participants (80.9%, n = 310/383) had not received formal education on NWFAs, which did not significantly differ across the practice approaches: 66.7% of solely weight-focused participants (n = 2/3); 70.6% of moderately weight focused (n = 36/51); 83.9% of combined approaches (n = 130/155); 81.0% of weight inclusive (n = 115/142); 84.4% of weight liberated (n = 27/32). Among those who practiced using NWFAs, the most common professional development activities undertaken included Craving Change (41.8%, n = 160/383) [25], Diabetes Educator Certification (31.3%, n = 120/383) [26], Dietitians of Canada and Obesity Canada—Obesity Learning Retreat (11.2%, n = 43/383) [27], Balanced View British Columbia (6.8%, n = 26/383) [28], The Strategic Centre for Obesity Professional Education (SCOPE) certification from the World Obesity Foundation (6.5%, n = 25/383) [29], informal learning from equity seeking groups or individuals holding diverse identities/experiences (6.0%, 23/383), and Body Image Training with Marci Evans (5.5%, 21/383) [30].

## 4. Discussion

This is the first Canadian study, and among the first globally, to characterize the different practice approaches used among dietitians when working with higher-weight adult clients, resulting in highly novel data on the use of NWFAs. As expected, weight loss and the recommendation of foods and dietary behaviours associated with weight loss were more common among dietitians who use WFAs. It is also no surprise that practice approaches that focus less on weight were more commonly used by participants whose practice aligned with NWFAs. Additionally, this study uniquely assessed nutrition therapy approaches and techniques and dietary assessment used in clinical settings by practice type. Finally, a highly novel and important contribution of this study is the description of each practice approach category that were derived and validated from the descriptive labels used by participants to characterize their practice approach. There are no known studies or policy documents that formally define NWFAs or compare nutrition therapy techniques used by dietitians who use different practice approaches.

This study found that 45.5% of Canadian dietitians practice using NWFAs. The proportion of Canadian dietitians using NWFAs is notably higher than the finding reported by Willer et al. (2019), where only 18.3% of Australian dietitians used a “weight-neutral” practice approach. Although misclassification of participants is a potential risk in survey research, the variation in findings may be influenced by the different methods to classify dietitians in these two studies. Willer et al. (2019) used five questions to classify dietitians into “weight centric”, “weight neutral” or “mixed approach” categories, considering their preference, current practice, and whether or not they correctly answered a knowledge-based question on HAES. In contrast, our study examined a spectrum of approaches that reflect noted variations within the WFAs (weight centric) and NWFAs (weight neutral) dichotomies, considering the importance participants placed on weight loss, body weight as an indicator of health, views regarding calorie restriction, etc. Thus, our classification might be more sensitive to include some other participants because of this expanded set of characterizing features of NWFAs. As a result, fewer dietitians in our study were classified into the combination, or “mixed”, approach, compared to in the Willer et al. (2019) paper. There is other evidence that misclassification may not have occurred. As anticipated, our study observed statistically significant differences in the use of nutrition therapy recommendations and techniques between dietitians across the range of practice approaches explored. For instance, significantly fewer dietitians using NWFAs utilized weight-centric assessment practices (e.g., weighing clients). For many variables assessed in this study, the use of weight centric practices among dietitians using NWFAs was very low (e.g., almost no RDs recommend reducing caloric intake), but not always. Even Willer et al. (2019) identified a small proportion of “weight neutral” dietitians used weight-centric practices. It is important to recognize that these dietitians may be implementing practices that are consistent with their workplace policies and procedures (even if they are able to practice according to their desired approach, as many were able to do in this study), which unfortunately cannot be discerned from survey data. Likewise, nutrition therapy techniques that are ascribed to NWFAs, such as mindful eating and intuitive eating, are being used to promote weight loss by dietitians using WFAs. Additionally, we found that many dietitians who use NWFAs recognize the word “obesity”, when others do not, which may be considered a surprising and contradictory in nature. Overall, these findings reflect the diversity and fluidity of dietetic practice and may suggest that dietetic practice is not as “black and white” as one may perceive, even when the theoretical and philosophical underpinnings of the different approaches and practices (e.g., WFAs versus NWFAs) conflict. It would be highly beneficial for future research studies to more deeply explore these topics in qualitative research studies.

The only other Canadian data similar to this topic were from a 2004 survey of Canadian dietitians, which found that most believed that healthy eating habits should be emphasized over calorie reduction [17]. However, the survey used did not assess dietetic practice across a range of approaches that consider the importance of weight loss when working with higher-weight clients [17]. The relatively large proportion of Canadian dietitians using NWFAs in their practice may be driven by efforts to minimize the adverse effects of weight stigma which is encouraged by the Canadian Adult Obesity CPGs and other non-governmental organizations. For instance, Dietitians of Canada signed in 2020 the Joint International Consensus Statement to signal their commitment to ending weight stigma by reducing the focus on weight-based messages [31]. However, we note that some organizations have co-opted the use of NWFAs with language that may not genuinely follow the philosophy of these approaches, and subsequently dietitians may be influenced while receiving training from these organizations. The high proportion of Canadian dietitians using NWFAs may also be driven by recent research showing that a focus on weight loss increases social isolation, depression, disordered eating, cortisol levels, non-adherence to medications, and metabolic syndrome [7,32,33,34,35]. Newer approaches to behavioural lifestyle changes that are more clinically effective at facilitating changes of health behaviours and target reducing weight stigma are needed [4,16,36].

Previous studies have primarily used qualitative methods with small sample sizes to explore the use of NWFAs among dietitians [37,38]. This study is among the first to use quantitative methods, which are generalizable and adequately powered to describe and examine the prevalence of NWFAs used among dietitians. The quantitative, cross-sectional study design was made possible by carefully constructing and validating a classification framework comprising five practice approach categories, which participants could then identify as their preferred approach. Participant responses also enabled further refinement of the practice approach categories and discernment of nuances in the nutrition therapy techniques used across the range of approaches. This finding highlights a knowledge gap that should be addressed in future research.

This study, and findings from the one other study to examine NWFAs [17], found that most participants had received no formal education on NWFAs, despite that a large proportion of participants used this approach. The lack of formal education/training and standardized guidelines for the implementation of NWFAs has implications for practice safety and quality of client care. Without training and evidence-informed guidelines, there is a risk that dietitians will misinterpret the use of NWFAs in their practice and not effectively use NWFAs with their clients. Our study found that 43% of participants who used NWFAs reported implementing “other” nutrition therapy recommendations outside of Canadian health eating policy, CPGs, or common techniques identified in the literature or used by the numerous dietitians (who use NWFAs in practice) who were consulted in the development and validation of the questionnaire. This finding demonstrates that we could not fully characterize how dietitians using NWFAs are practicing, and it shows that diverse and uncharacterized nutrition therapy approaches are commonly being used. It also further highlights the largely unexplored nature of this topic and the need for further research. Indeed, a lack of dietetic training, best practice guidelines, and limited continuing education opportunities may contribute to such high variability in practice techniques used by dietitians when implementing NWFAs; these should be prioritized by educational institutions and dietetic organizations.

The gap between education and training and the practice approaches being used by dietitians reported in this study is supported by other research demonstrating that dietitians hold positive attitudes towards nutrition therapy techniques commonly associated with NWFAs, such as intuitive eating [19]. However, many dietitians feel that they have insufficient training to do so [19]. One study reported that some who receive training on “obesity” management and counselling at the undergraduate level would like the scope of the curriculum on this topic to be expanded [17]. Collectively, these data indicate that education and training programs must better prepare dietitians to implement practice approaches beyond WFAs. Increasing education and training opportunities could be achieved in the short-term by including NWFAs as a standard component in undergraduate dietetic curriculums and as a core competency in dietetic practicum rotations. In the long-term, revising the Integrated Competencies for Dietetic Education and Practice, hiring faculty with experience in using NWFAs, offering professional development opportunities, and advocacy by professional associations is also needed to ensure adequate training for dietitians in NWFAs. Given that there is no widely accepted definition of NWFAs, developing a clinical care pathway that describes NWFAs for dietitians and how best to implement NWFAs is necessary. These definitions will continue to evolve as newer research becomes available. More research and resources are needed to help guide dietetic educators to reflect how dietitians are currently practicing with higher-weight adult clients. This study focused on exploring adult higher-weight clients, it would be helpful for further research to explore practice approaches dietitians are using with higher-weight pediatric and adolescent populations as well.

Our sample included a large proportion of participants who identified as White women, which reflects the demographic profile of the dietetic profession in Canada that has been reported elsewhere [39,40]. Data from the 2016 Accreditation Council for Education in Nutrition and Dietetics showed that among those enrolled in dietetics education programs 69.3% were Caucasian and 5.5% were Black (non-Hispanic) [41]. Caucasian undergraduates in nutrition programs, compared to non-Caucasian, are 4-fold more likely to become licensed as a Registered Dietitian in Canada [40,42]. Further education may be needed as practice approaches evolve in dietetics.

### Limitations

One limitation of this study is the relatively high proportion of participants from Ontario and Alberta although these provinces have large populations in Canada, so this may reflect the true distribution of dietitians across the country. Although recruitment through social media may introduce sampling bias, the survey was also distributed to all registrants of dietetic regulatory colleges in five provinces, and to distribution lists of universities, which allowed the survey invitation to be distributed to dietitians who might not be reached via social media. Additionally, the survey was only available in English. Although English is the language spoken by most Canadians, Canadian dietitians who exclusively speak French were excluded. The COVID-19 pandemic may also have impacted sampling in this study because some dietitians were seconded to non-clinical roles and may have been preoccupied with other responsibilities or had limited access to email. Finally, a previous study observed limited knowledge of weight inclusive practice goals among dietitians, which could contribute to participants incorrectly identifying with a practice approach if they are unfamiliar with the terms and definitions [3]. However, the practice approaches in the present study were labelled with letters (A–E) so that participants had to read and identify which the practice approach descriptions and choose the one that most reflected their practice, which reduced any potential response bias.

## 5. Conclusions

This is among the first studies in Canada, and globally, to broadly examine what practice approaches dietitians are using with higher-weight adult clients, by examining dietetic practice across a spectrum of five practice approaches based on how important dietitians perceive weight and weight loss. This study generated highly novel data that provide more systematic and thorough descriptions of the practice approaches that vary with respect to emphasis on body weight and weight loss that are used by Canadian dietitians. Almost half of dietitians solely use NWFAs in their dietetic practice, with another 41% using a combination of WFAs and NWFAs. This study also found that most dietitians have no formal education or training in NWFAs. This research provides a basis for future research exploring NWFAs and for advocacy efforts to enable a dietetic curriculum to evolve to meet the needs of practicing dietitians.

## Figures and Tables

**Table 1:** Practice approach classification framework ^a^.

Practice Approach	Description
(A) Solely weight-focused	Body weight is an important indicator of health status and is usually measured at each visit. Ways of eating promote weight loss, sometimes regardless of body size. Nutrition therapy focuses on calorie reduction (“calories in/calories out”), and possibly diet quality and eating patterns.
(B) Moderately weight-focused	Body weight is usually viewed as an important indicator of health status and is usually measured at each visit. Obesity is viewed as a risk factor for disease or as a chronic disease itself. Usually includes weight loss as an outcome. Nutrition therapy focuses on calorie reduction, and possibly diet quality and eating patterns.
(B) Combination	Fluctuate between weight-focused and weight-inclusive approaches within practice Body weight is usually not an important indicator of health status. Recognizes that obesity is a chronic disease. Usually does not focus on weight change or loss as an outcome. Nutrition therapy focuses on diet quality and eating patterns.
(D) Weight inclusive	Weight is not a measured outcome or an outcome to be achieved. Obesity is not discussed as a factor contributing to chronic disease. Obesity may not be recognized as a term. Discusses weight with clients out of client interest but understands weight as a normal part of body diversity. Nutrition therapy focuses on diet quality and eating patterns.
(E) Weight liberated	Weight is not a measured outcome or an outcome to be achieved. Obesity is not recognized as a term. Discusses weight with clients out of client interest but understands weight as a normal part of body diversity. Does not take a healthism approach (healthism is the preoccupation with personal health as the primary focus of well-being, usually obtained through modifying lifestyle behaviours). Recognizes that diet is an outcome of inequity and social justice and advocates for and/or works upstream to deconstruct systemic inequity issues.

^a^ Participants were blinded to the approach name, by referring to the approaches A–E, to reduce the potential for a biased classification.

**Table 2:** Participant demographic characteristics.

	Practice Approaches						*p* Value ^a^
	All (n = 383)	Solely Weight Focused (n = 3)	Moderately Weight Focused (n = 51)	Combination (n = 155)	Weight Inclusive (n = 142)	Weight Liberated (n = 32)	
**Gender**							
Woman	363 (94.8)	2 (0.6)	45 (12.4)	149 (41.0)	139 (38.3)	28 (7.7)	<0.001
Man	11 (2.9)	1 (9.1)	5 (45.5)	3 (27.3)	2 (18.2)	0 (0.0)	
Non-binary	1 (0.3)	0 (0.0)	0 (0.0)	0 (0.0)	0 (0.0)	1 (100.0)	
Prefer not to answer	8 (2.0)	0 (0.0)	1 (12.5)	3 (37.5)	1 (12.5)	2 (25.0)	
**Race ^b^**							
White, e.g., European	315 (82.2)	1 (0.3)	38 (12.1)	126 (40.0)	124 (39.4)	26 (8.3)	0.061
East Asian, e.g., Chinese, Korean	29 (7.6)	0 (0.0)	3 (10.3)	13 (44.8)	10 (34.5)	3 (10.3)	
Other, e.g., Middle Eastern, Latin American, Indigenous	27 (7.0)	1 (3.7)	6 (22.2)	11 (40.7)	6 (22.2)	3 (11.1)	
South/Southeast Asian	12 (3.1)	1 (8.3)	4 (33.3)	5 (41.7)	2 (16.7)	0 (0.0)	
**Province and Territories ^c^**							
Ontario	154 (40.2)	1 (0.6)	17 (11.0)	63 (41.0)	60 (39.0)	13 (8.4)	0.037
Alberta	120 (31.3)	1 (0.8)	25 (20.8)	53 (44.2)	34 (28.3)	7 (5.8)	
British Columbia	35 (9.1)	0 (0.0)	2 (5.7)	11 (31.4)	19 (54.3)	3 (8.6)	
Saskatchewan	17 (4.4)	0 (0.0)	0 (0.0)	8 (47.1)	9 (53.0)	0 (0.0)	
Quebec	14 (3.7)	0 (0.0)	2 (14.3)	1 (7.1)	6 (42.9)	5 (35.7)	
Manitoba	14 (3.7)	0 (0.0)	3 (21.4)	5 (35.7)	4 (28.6)	2 (14.3)	
New Brunswick	12 (3.1)	1 (8.3)	1 (8.3)	7 (58.3)	2 (16.7)	1 (8.3)	
Nova Scotia	9 (2.3)	0 (0.0)	1 (11.1)	4 (44.4)	3 (33.3)	1 (11.1)	
Newfoundland and Labrador	4 (1.0)	0 (0.0)	0 (0.0)	2 (50.0)	2 (50.0)	0 (0.0)	
Prince Edward Island	3 (0.8)	0 (0.0)	0 (0.0)	1 (33.3)	2 (66.7)	0 (0.0)	
Northwest Territories	1 (0.3)	0 (0.0)	0 (0.0)	0 (0.0)	1 (100.0)	0 (0.0)	
**Years of Practice as a Registered Dietitian**							
0–5	120 (31.3)	2 (1.7)	15 (12.5)	43 (35.8)	48 (40.0)	12 (10.0)	0.973
6–10	86 (22.5)	0 (0.0)	11 (12.8)	37 (43.0)	32 (37.2)	6 (7.0)	
11–15	76 (19.8)	1 (1.3)	9 (11.8)	32 (42.1)	26 (34.2)	8 (10.5)	
16–20	48 (12.5)	0 (0.0)	6 (12.5)	21 (44.0)	19 (39.6)	2 (4.2)	
>20	53 (13.8)	0 (0.0)	10 (18.9)	22 (41.5)	17 (32.1)	4 (7.5)	
**Highest Level of Education**							
Bachelor’s	274 (71.5)	2 (0.7)	41 (15.0)	105 (38.3)	100 (36.5)	26 (9.5)	0.315
Master’s	96 (25.0)	1 (1.0)	9 (9.4)	43 (44.8)	39 (40.6)	4 (4.2)	
Other	13 (3.4)	0 (0.0)	1 (7.7)	7 (53.8)	3 (23.1)	2 (15.4)	
**Primary Area of Practice**							
Primary care	195 (50.9)	0 (0.0)	17 (8.7)	76 (39.0)	81 (41.5)	21 (10.8)	<0.001
Outpatient	126 (32.9)	3 (2.4)	26 (20.6)	55 (43.7)	40 (31.7)	2 (1.6)	
Other	62 (16.2)	0 (0.0)	8 (12.9)	24 (38.7)	21 (33.9)	9 (14.5)	
**Type of Community**							
Urban	208 (54.3)	2 (1.0)	35 (16.8)	73 (35.1)	78 (37.5)	20 (9.6)	0.192
Suburban	90 (23.5)	1 (1.1)	7 (7.8)	49 (54.4)	30 (33.3)	3 (3.3)	
Rural	65 (17.0)	0 (0.0)	7 (10.8)	26 (40.0)	26 (40.0)	6 (9.2)	
Remote	4 (1.0)	0 (0.0)	0 (0.0)	2 (50.0)	2 (50.0)	0 (0.0)	
Do not work in a single community	16 (4.2)	0 (0.0)	2 (12.5)	5 (31.3)	6 (37.5)	3 (18.8)	

Categorical data are presented as the frequency (n) and the percent (%). ^a^ Fisher’s exact test was used to assess any differences between practices approaches. ^b^ Additionally included as other race categories, but not listed, were Arab, Iranian, Afghan, Egyptian, Lebanese, Turkish, and Kurdish; Black, e.g., African Caribbean and Black; Latinx, e.g., Latin American and Hispanic; Indigenous, e.g., First Nations and Métis, Inuk (Inuit). ^c^ There were no responses from participants from Nunavut or the Yukon Territories.

**Table 3:** The proportion of dietitians who practiced according to each practice approach and the descriptive labels used to characterize each practice approach.

Approach	n (%)	Descriptive Labels Participants Used to Characterize Their Practice Approach ^a^
Solely weight focused	3 (0.8%)	Patient led
Moderately weight focused	51 (13.3%)	Patient/client centred, lifestyle and behaviour focused, individualized dietary behaviours, flexible, health centred, goal focused, inclusive, supportive and directive, weight management
Combination	155 (40.5%)	Non-diet, patient/client centred, health focused, behaviour focused, weight neutral, HAES informed, weight inclusive informed, intuitive eating informed, modifying weight, non-weight focused, best weight, obesity management
Weight inclusive	142 (37.1%)	Healthism, weight inclusive, normalized eating, anti-diet, HAES, client/patient focused, inclusive, intuitive eating, non-judgmental, non-diet, strength based, self-compassion, eating skills, body liberation, humanist, body diversity, mindful eating
Weight liberated	32 (8.3%)	Weight inclusive, non-diet, fat positive, HAES, social justice oriented, anti-oppressive, trauma informed, patient/client focused, values based, well-being focused, body diversity, lived experience, anti-diet

^a^ Data based on a content analysis of open-ended questions. Some participants included more than one label in their description. HAES = Health At Every Size^®^.

**Table 4:** Definitions of obesity by practice approach.

	Practice Approaches						*p* Value ^a^
	All (n = 383)	Solely Weight Focused (n = 3)	Moderately Weight Focused (n = 51)	Combination (n = 155)	Weight Inclusive (n = 142)	Weight Liberated (n = 32)	
A complex and progressive chronic disease	194 (50.7)	2 (66.7)	32 (62.7)	106 (68.4)	52 (36.6)	2 (6.2)	<0.001
Characterized by abnormal, excessive body fat (adiposity) that impairs health	186 (48.6)	2 (66.7)	36 (70.6)	89 (57.4)	55 (38.7)	4 (12.5)	
Body mass index [BMI] >30 kg/m^2^	104 (27.2)	2 (66.7)	24 (47.0)	45 (29.0)	30 (21.1)	3 (9.4)	
I do not recognize or use this language	102 (26.6)	0 (0.0)	0 (0.0)	8 (5.2)	66 (46.5)	28 (87.5)	

^a^ Fisher’s exact test was used to assess any differences between practices approaches. Categorical data are presented as the frequency (n) and the percent (%).

**Table 5:** Nutrition assessment methods, nutrition therapy approaches, and techniques commonly used by participants, overall and by practice approach.

	Practice Approaches ^b^						*p* Value ^a^
	All (n = 383)	Solely Weight Focused (n = 3)	Moderately Weight Focused (n = 51)	Combination (n = 155)	Weight Inclusive (n = 142)	Weight Liberated (n = 32)	
**Assessment**							
Monitor health behaviours (e.g., diet and exercise) as an indicator of changed health risk	367 (95.8)	3 (100.0)	49 (96.1)	150 (96.8)	138 (97.0)	27 (84.4)	0.048
Assess metabolic parameters (lipid profile, blood glucose, liver enzymes, vitamin and mineral status, etc.)	366 (95.6)	3 (100.0)	48 (94.1)	150 (96.8)	137 (96.5)	28 (87.5)	0.194
Assess mental health status (e.g., depression, addition, and eating disorders)	360 (94.0)	3 (100.0)	47 (92.2)	145 (93.5)	134 (94.4)	31 (96.9)	0.860
Assess social health (e.g., social support, connection to care givers, and living conditions)	350 (91.4)	3 (100.0)	41 (80.4)	146 (94.2)	130 (91.5)	30 (93.8)	0.075
Assess financial health by collecting economic information, including food security	314 (82.0)	3 (100.0)	41 (80.4)	129 (83.2)	111 (78.2)	30 (93.8)	0.275
Assess mechanical health (e.g., back pain, osteoarthritis, and sleep apnea)	308 (80.4)	2 (66.7)	39 (76.5)	121 (78.1)	117 (82.4)	29 (90.6)	0.341
Weighs clients	150 (39.2)	3 (100.0)	43 (84.3)	71 (45.8)	28 (19.7)	5 (15.6)	<0.001
Calculate body mass index (BMI) to assess health risk	142 (37.1)	2 (66.7)	41 (80.4)	71 (45.8)	26 (18.3)	2 (6.3)	<0.001
Measure body composition	32 (8.4)	0 (0.0)	8 (15.7)	17 (11.0)	7 (4.9)	0 (0.0)	0.042
Other	52 (13.6)	0 (0.0)	5 (9.8)	17 (11.0)	21 (14.8)	9 (28.1)	0.130
**Nutrition Therapy Approaches**							
Increasing fruits and vegetables	364 (95.0)	3 (100.0)	46 (90.2)	151 (97.4)	138 (97.2)	26 (81.3)	0.014
Increasing intake of whole grains	338 (88.3)	3 (100.0)	40 (78.4)	141 (91.0)	130 (91.5)	24 (75.0)	<0.001
Increasing dietary variety	333 (86.9)	3 (100.0)	39 (76.5)	135 (87.1)	127 (89.4)	29 (90.6)	0.215
Increasing intake of pulses	328 (85.6)	2 (66.7)	35 (68.6)	141 (91.0)	125 (88.0)	25 (78.1)	<0.001
Doing more physical activity	317 (82.8)	3 (100.0)	46 (90.2)	133 (85.8)	113 (79.6)	22 (68.8)	0.130
Alternative foods for snacking	269 (70.2)	3 (100.0)	42 (11.0)	123 (32.1)	89 (23.2)	12 (3.1)	<0.001
Replacing saturated/trans fats with unsaturated fats	246 (64.2)	3 (100.0)	25 (49.0)	113 (72.9)	89 (62.7)	16 (50.0)	0.004
Mediterranean dietary pattern	211 (55.1)	1 (33.3)	27 (52.9)	89 (57.4)	85 (59.9)	9 (28.1)	0.963
Canada’s Food Guide	206 (53.8)	1 (33.3)	23 (45.1)	89 (57.4)	81 (57.0)	12 (37.5)	0.018
Low-glycemic index dietary pattern	160 (41.8)	2 (66.7)	17 (33.3)	79 (51.0)	55 (38.7)	7 (21.9)	0.405
Dietary Approaches to Stop Hypertension dietary pattern (DASH)	128 (33.4)	0 (0.0)	17 (33.3)	58 (37.4)	46 (32.4)	7 (21.9)	0.476
Modifying specific macronutrients, e.g., low carbohydrate and high protein	112 (29.2)	2 (66.7)	26 (51.0)	49 (31.6)	32 (22.5)	3 (9.4)	0.077
Intake of certain foods to reduce calories	95 (24.8)	3 (100.0)	32 (62.7)	45 (29.0)	14 (9.9)	1 (3.1)	<0.001
Reducing overall caloric intake	50 (13.1)	2 (66.7)	25 (49.0)	21 (13.5)	2 (1.4)	0 (0.0)	<0.001
Reducing total fat intake	49 (12.8)	2 (66.7)	17 (33.3)	21 (13.5)	8 (5.6)	1 (3.1)	0.003
Vegetarian dietary pattern	46 (12.0)	0 (0.0)	6 (11.8)	18 (11.6)	19 (13.4)	3 (9.4)	0.006
Time-limited feeding, i.e., intermittent fasting	23 (6.0)	0 (0.0)	8 (15.7)	12 (7.7)	3 (2.1)	0 (0.0)	0.157
The Nordic dietary pattern	9 (2.3)	0 (0.0)	2 (3.9)	2 (1.3)	4 (2.8)	1 (3.1)	0.004
A ketogenic diet	6 (1.6)	0 (0.0)	3 (5.9)	2 (1.3)	1 (0.7)	0 (0.0)	<0.001
Other	69 (18.0)	0 (0.0)	5 (9.8)	19 (12.3)	31 (21.8)	14 (43.8)	<0.001
**Nutrition Therapy Techniques**							
Techniques of mindful eating	337 (88.0)	2 (66.7)	44 (86.3)	136 (87.7)	127 (89.4)	28 (87.5)	0.649
Recognize clients’ lived experiences impact their lives in ways that are often hidden to providers	304 (79.4)	2 (66.7)	32 (62.7)	115 (74.2)	124 (87.3)	31 (96.9)	<0.001
Discuss the structural barriers to their being/feeling healthy or well	289 (75.5)	2 (66.7)	38 (74.5)	113 (72.9)	108 (76.1)	28 (87.5)	0.433
Principles of intuitive eating	271 (70.8)	0 (0.0)	15 (29.4)	102 (65.8)	124 (87.3)	30 (93.8)	<0.001
Principles of compassion-informed counselling	250 (65.3)	3 (100.0)	31 (60.8)	92 (59.4)	96 (67.6)	28 (88.0)	0.015
Principles of Health At Every Size^®^	248 (64.8)	1 (33.3)	13 (25.5)	84 (54.2)	121 (85.2)	29 (90.6)	<0.001
Recommend keeping a hunger awareness diary	239 (62.4)	1 (33.3)	25 (49.0)	98 (63.2)	93 (65.5)	22 (68.8)	0.192
Recommend that clients do not weigh themselves	206 (53.8)	0 (0.0)	14 (27.5)	81 (52.3)	84 (59.2)	27 (84.4)	<0.001
Principles of culturally safe care	192 (50.1)	1 (33.3)	12 (23.5)	83 (53.5)	72 (50.7)	24 (75.0)	<0.001
Recommend keeping a food intake diary	183 (47.8)	3 (100.0)	37 (72.5)	93 (60.0)	45 (31.7)	5 (15.6)	<0.001
Principles of harm reduction counselling	162 (42.3)	0 (0.0)	15 (29.4)	59 (38.1)	63 (44.4)	25 (78.1)	<0.001
Recommend eating smaller, more frequent meals	136 (35.5)	1 (33.3)	25 (49.0)	68 (43.9)	39 (27.0)	3 (9.4)	<0.001
Principles of trauma-informed counselling	121 (31.6)	0 (0.0)	9 (17.6)	42 (27.1)	48 (33.8)	22 (68.8)	<0.001
Draw on equity-seeking clients’ experiences of oppression	93 (24.3)	0 (0.0)	5 (9.8)	28 (18.1)	36 (25.4)	24 (75.0)	<0.001
Recommend limiting snacking	81 (21.1)	2 (66.7)	25 (49.0)	43 (27.7)	10 (7.0)	1 (3.1)	<0.001
Recommend weight loss	20 (5.2)	2 (66.7)	12 (23.5)	6 (3.9)	0 (0.0)	0 (0.0)	<0.001
Recommend keeping a weight diary	15 (3.9)	1 (33.3)	11 (21.6)	3 (1.9)	0 (0.0)	0 (0.0)	<0.001
Recommend commercial weight loss products	1 (0.3)	0 (0.0)	1 (2.0)	0 (0.0)	0 (0.0)	0 (0.0)	0.219
Other	26 (6.8)	0 (0.0)	3 (5.9)	9 (5.8)	10 (7.0)	4 (12.5)	0.635

^a^ Fisher’s exact test for count data with stimulated *p*-value (based on 2000 replicates). ^b^ Data are represented as comparing “Yes” versus “No” responses for each variable. Categorical data are presented as the frequency (n) and the percent (%).

## Data Availability

Data are available upon request from the corresponding author.
